# Stable kinetochore–microtubule attachment is sufficient to silence the spindle assembly checkpoint in human cells

**DOI:** 10.1038/ncomms10036

**Published:** 2015-12-01

**Authors:** Eric C. Tauchman, Frederick J. Boehm, Jennifer G. DeLuca

**Affiliations:** 1 Cell and Molecular Biology Program, Colorado State University, Fort Collins, Colorado 80523, USA; 2 Department of Statistics, University of Wisconsin-Madison, Madison, Wisconsin 53706, USA; 3 Department of Biochemistry and Molecular Biology, Colorado State University, Fort Collins, Colorado 80523, USA

## Abstract

During mitosis, duplicated sister chromatids attach to microtubules emanating from opposing sides of the bipolar spindle through large protein complexes called kinetochores. In the absence of stable kinetochore–microtubule attachments, a cell surveillance mechanism known as the spindle assembly checkpoint (SAC) produces an inhibitory signal that prevents anaphase onset. Precisely how the inhibitory SAC signal is extinguished in response to microtubule attachment remains unresolved. To address this, we induced formation of hyper-stable kinetochore–microtubule attachments in human cells using a non-phosphorylatable version of the protein Hec1, a core component of the attachment machinery. We find that stable attachments are sufficient to silence the SAC in the absence of sister kinetochore bi-orientation and strikingly in the absence of detectable microtubule pulling forces or tension. Furthermore, we find that SAC satisfaction occurs despite the absence of large changes in intra-kinetochore distance, suggesting that substantial kinetochore stretching is not required for quenching the SAC signal.

## Abstract (web summary)

The spindle assembly checkpoint prevents mitotic progression when chromosomes are not properly attached to the mitotic spindle. Here Tauchman *et al.* show that stable microtubule attachment to the kinetochore, and not tension generated from this interaction, is sufficient to silence the checkpoint.

## Graphical abstract

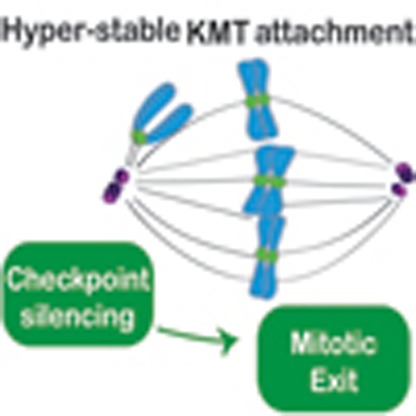

Accurate segregation of duplicated chromosomes in mitosis is critical for the viability of daughter cells and for the maintenance of genomic integrity. Incorrect chromosome segregation can result in aneuploidy, a condition associated with tumorigenesis and developmental defects^1^. On mitotic entry, dynamic microtubules form a bipolar spindle, which is responsible for capturing and congressing mitotic chromosomes. These events require proper attachment between spindle microtubule plus ends and kinetochores, large protein structures built on centromeric chromatin^2^,^3^. In order for cells to successfully complete mitosis, chromosomes must congress to the spindle equator and generate amphitelic kinetochore attachments, in which each sister kinetochore is connected to microtubules from each of the two opposite poles. In the absence of such attachments the cell will delay mitotic exit. The mechanism that monitors and responds to kinetochore–microtubule attachment is the spindle assembly checkpoint (SAC). In the presence of unattached kinetochores, SAC proteins form a complex that inhibits the anaphase promoting complex/cyclosome by binding to its activator, Cdc20 (refs 4, 5, 6, 7). Precisely how the inhibitory SAC signal is extinguished in response to microtubule binding remains unresolved, although both the physical engagement of microtubules with core kinetochore–microtubule attachment factors and the ensuing tension that follows are considered to be important aspects of the signalling process^8^,^9^.

In the case of correctly attached bi-oriented sister kinetochore pairs, kinetochore microtubules are stabilized, at least in part, in response to a decrease in Aurora B kinase phosphorylation of outer kinetochore substrates including Hec1/Ndc80 and KNL1 (refs 10, 11). Decreased phosphorylation of these substrates results in kinetochore–microtubule stabilization, development of inter-kinetochore tension, and SAC silencing^4^,^6^,^12^,^13^. Although it is well-accepted that kinetochore tension develops after formation of bi-oriented kinetochore–microtubule attachments, there is also evidence that tension itself can impact kinetochore–microtubule stability^14^. Classic experiments in grasshopper spermatocytes demonstrated that pulling on kinetochores with a microneedle resulted in kinetochore–microtubule stabilization^15^. More recently it was shown that syntelic kinetochore–microtubule attachments can be stabilized in *Drosophila* cells by experimentally increasing polar ejection forces, and thereby increasing kinetochore tension^16^. Finally, application of tension to purified budding yeast kinetochores has been shown to activate a ‘catch-bond' mechanism that directly stabilizes microtubule attachment^17^.

It is clear that kinetochore–microtubule attachments can be stabilized by changes in kinetochore kinase activity and by application of tension, and in cells, these two mechanisms likely work together to increase kinetochore–microtubule stability^14^. An issue that still remains unresolved, however, is whether the presence of stable kinetochore microtubules is sufficient to induce changes in the kinetochore that lead to SAC silencing, or if kinetochore tension is additionally required. This issue has been difficult to address, since on chromosome bi-orientation and formation of correct kinetochore–microtubule attachments the development of kinetochore tension is a consequence. Despite this, there is evidence that microtubule attachment itself is sufficient for SAC silencing. In a landmark study by the Rieder lab using PtK1 cells, a single remaining unattached kinetochore was laser ablated, which resulted in silencing the SAC and entry into anaphase^18^. In this case, tension between the two sister kinetochores (typically monitored by the distance between kinetochores) was surely lost, pointing to stable microtubule attachment as the critical parameter monitored by the SAC. However, it is likely that the remaining kinetochore was still under tension, resulting from pulling forces produced by the attached microtubules and pushing forces produced from polar ejection forces^16^,^19^,^20^. A later study demonstrated that the addition of taxol, which resulted in loss of inter-kinetochore tension, but retention of stable kinetochore–microtubule attachment, resulted in eviction of the SAC protein Mad2 from kinetochores in PtK1 cells^21^, providing further support for the idea that stable attachment is sufficient to silence the SAC. Similar to the laser ablation study, it is likely that in the presence of taxol, individual kinetochores remained under tension^22^. This is important to consider, since recent studies have suggested that tension within individual kinetochores, detected by displacement of outer kinetochore components from the inner kinetochore (referred to as ‘intra-kinetochore stretching'), on microtubule attachment is the signal detected by the SAC machinery to silence the checkpoint and initiate anaphase^23^,^24^. Although intra-kinetochore distance increases on microtubule attachment and is indeed correlated to SAC satisfaction^23^,^24^,^25^, it remains to be determined if intra-kinetochore stretching serves as the critical signal for SAC silencing. Alternatively, increased intra-kinetochore distances may result from changes in kinetochore architecture that are a consequence of stable kinetochore–microtubule attachment, which ultimately signals to quench SAC activation^19^,^20^.

Here we investigate how hyper-stabilization of kinetochore–microtubule attachment affects progression through mitosis and SAC satisfaction in the absence of chromosome bi-orientation. To induce kinetochore–microtubule hyper-stabilization, we used a mutant version of the kinetochore–microtubule attachment factor Hec1 that is unable to be phosphorylated by Aurora B kinase: 9A-Hec1, in which nine identified Aurora B target sites were mutated to alanine (9A) (ref. 10). Our previous studies demonstrated that cells depleted of endogenous Hec1 and rescued with 9A-Hec1-GFP harbour hyper-stable kinetochore microtubules and exhibit an increased number of erroneous attachments^10^,^26^. Here we find that the hyper-stable kinetochore–microtubule attachments in cells expressing 9A-Hec1-GFP are sufficient to silence the SAC, even in the absence of chromosome bi-orientation or experimentally induced tension. In addition, we find that SAC silencing occurs in the absence of large changes in intra-kinetochore distance, suggesting that substantial intra-kinetochore stretching is not required for quenching the SAC signal.

## Results

### 9A-Hec1 cells with unaligned chromosomes satisfy the SAC

**Figure 1:**
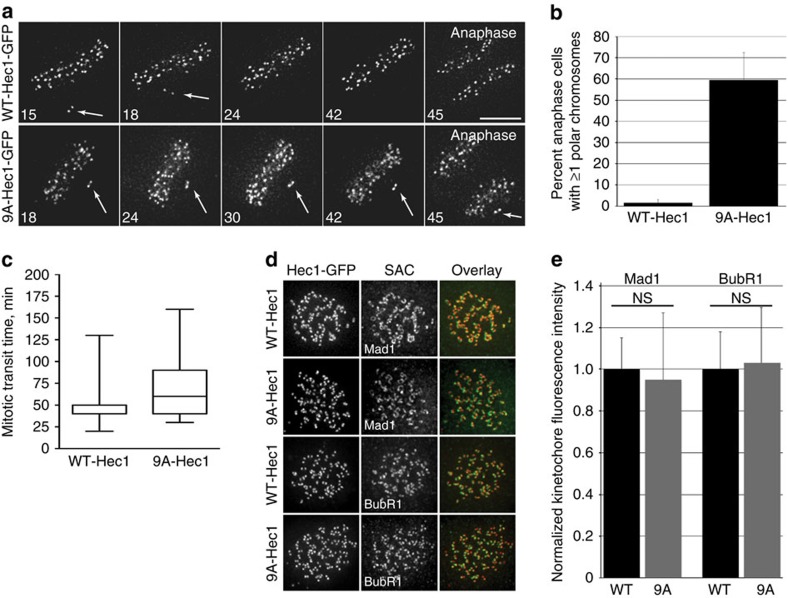
Cells expressing 9A-Hec1 satisfy the SAC and enter anaphase with pole-proximal chromosomes. (**a**) Time-lapse images of HeLa cells expressing WT- or 9A-Hec1-GFP. Cells expressing WT-Hec1-GFP enter anaphase only after all chromosomes are properly aligned at the metaphase plate. Cells expressing 9A-Hec1-GFP enter anaphase in the presence of polar, unaligned chromosomes. Arrows point to pole-proximal chromosomes. In the WT-Hec1-GFP-expressing cell shown, the pole-proximal chromosome eventually migrates to the metaphase plate. In the 9A-Hec1-GFP-expressing cell, the pole-proximal chromosome remains at the spindle pole, even after anaphase onset. Time, post-nuclear envelope breakdown, is shown in minutes. Scale bar, 5 μm. (**b**) Frequency of anaphase onset with pole-proximal chromosomes in WT- and 9A-Hec1-GFP-expressing cells. In all, 109 and 60 cells were scored, respectively, from three independent experiments. (**c**) Mitotic durations for WT- and 9A-Hec1-GFP-expressing cells. Mitotic duration was scored from cell rounding to cell cleavage. Average time in minutes is shown. *n*=100 cells for each condition. (**d**) Immunofluorescence images and (**e**) quantification of kinetochore fluorescence intensities of Mad1 (*n*=438 kinetochores for WT-Hec1-GFP-expressing cells; *n*=444 kinetochores for 9A-Hec1-GFP-expressing cells) and BubR1 (*n*=414 kinetochores for WT- and *n*=416 kinetochores for 9A-Hec1-GFP-expressing cells) from three independent experiments. Cells were treated with 5 μM nocodazole for 5 h. Error bars indicate standard deviation. NS=not significantly different, *P*≥0.01, as evaluated by Student's *t*-test (Mad1, *P*=0.66, BubR1, *P*=0.92).

The kinetochore protein Hec1/Ndc80 directly links kinetochores to microtubules in metazoans^27^,^28^. We previously demonstrated that cultured vertebrate cells expressing a mutant Hec1 that cannot be phosphorylated by Aurora B kinase on its disordered ‘tail' domain (9A-Hec1) generate hyper-stable kinetochore–microtubule attachments as evidenced by: (i) increased inter-kinetochore distances, (ii) thicker kinetochore fibres and (iii) an accumulation of syntelic attachments, in which both sister kinetochores of a pair are attached to a single pole^10^,^26^,^29^,^30^. To determine if these latter incorrect attachments are sufficient to satisfy the SAC, we time-lapse imaged HeLa cells inducibly expressing GFP-labelled 9A-Hec1 or wild-type (WT)-Hec1 (Supplementary Fig. 1a). Indeed, the majority of cells expressing 9A-Hec1 entered anaphase in the presence of one or more pole-associated, syntelically attached chromosomes (Fig. 1a–c and Supplementary Movies 1 and 2).

To determine if 9A-Hec1-expressing cells enter anaphase as a consequence of SAC defects, we quantified SAC protein levels at kinetochores following nocodazole-mediated microtubule depolymerization. Cells expressing 9A-Hec1 recruited equivalent levels of Mad1 and BubR1 to kinetochores as WT cells (Fig. 1d,e). Furthermore, 9A-Hec1-expressing cells exhibited a robust mitotic arrest after incubation in 5 μM nocodazole that was indistinguishable from cells expressing WT-Hec1 (Supplementary Fig. 2b,c and Supplementary Movies 3 and 4). We repeated these experiments under conditions in which endogenous Hec1 was depleted by RNAi and found identical results (Supplementary Figs 1b and 2b,d). We next determined whether the kinetics of SAC satisfaction were similar in 9A-Hec1 and WT-Hec1-expressing cells in the absence of microtubules. For this experiment, we treated cells expressing either WT- or 9A-Hec1 with nocodazole to depolymerize all microtubules and subsequently incubated cells in reversine, a small molecule inhibitor of Mps1, which is known to induce rapid SAC abrogation^31^. As shown in Supplementary Fig. 2, the kinetics of mitotic exit on reversine treatment between the two cell lines were indistinguishable. We conclude from these experiments that 9A-Hec1-expressing cells are not defective in SAC signalling, but progress through mitosis with pole-associated chromosomes as a consequence of SAC satisfaction.

### Stable kinetochore–microtubule attachment silences the SAC

**Figure 2:**
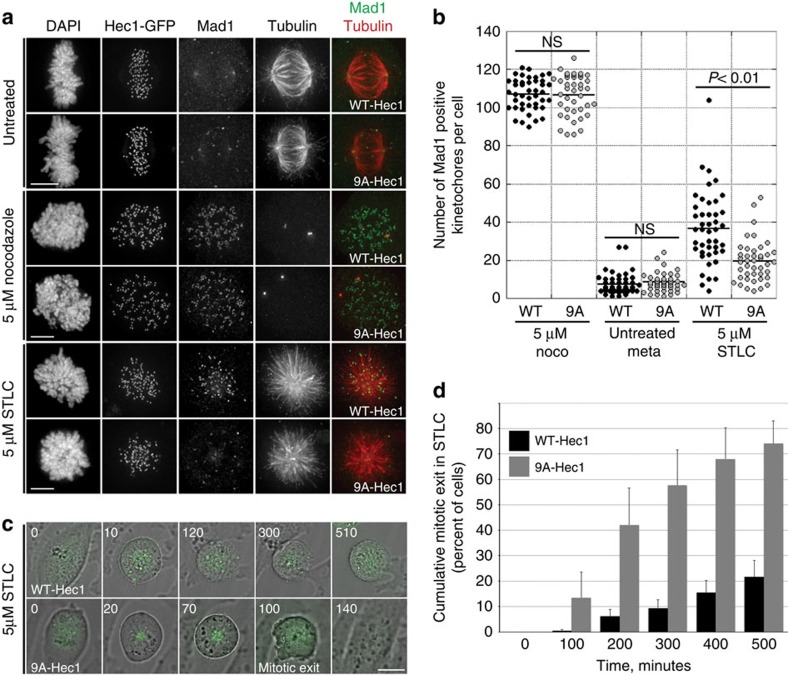
Stable kinetochore–microtubule attachment is sufficient to satisfy the SAC in the absence of chromosome bi-orientation. (**a**) Immunofluorescence images of cells expressing either WT- or 9A-Hec1-GFP, treated as indicated. Scale bars are 5 μm. (**b**) Quantification of Mad1-positive kinetochore staining. For the untreated (no drug) condition, only metaphase cells were scored. NS=not significantly different, *P*≥0.01, as evaluated by Student's *t*-test (5 μM nocodazole-treated cells, *P*=0.88; untreated cells, *P*=0.39). For each condition, at least 41 cells were scored from three experiments. (**c**) Stills from time-lapse imaging of STLC-treated WT- and 9A-Hec1-GFP-expressing cells. Shown are overlays of phase contrast and GFP images. Time is indicated in minutes, and the time of mitotic exit (as evidenced by membrane blebbing and chromosome decondensation) is also indicated. Scale bar, 5 μm. (**d**) Quantification of mitotic exit time for WT- and 9A-Hec1-GFP-expressing cells. Graph indicates cumulative mitotic exit at the indicated time point. Data from three independent experiments are included, *n*=345 cells for WT- and *n*=212 cells for 9A-Hec1-GFP-expressing cells. Error bars indicate s.d.

To further investigate the link between SAC satisfaction and stable kinetochore–microtubule attachment in the absence of pulling forces from chromosome bi-orientation, we created conditions whereby cells entirely lack proper kinetochore–microtubule attachments. Cells with monopolar spindles, generated by inhibition of Eg5 with *S*-trityl-L-cysteine (STLC), contain a large number of monotelic and syntelic attachments^32^,^33^,^34^. Consequently, cells mount a mitotic arrest due to the activity of the Aurora B kinase-mediated error-correction machinery, which destabilizes incorrectly attached microtubules^32^,^33^. STLC-treated cells expressing WT-Hec1 largely arrested in mitosis owing to the presence of a large number of unattached kinetochores, as evidenced by retention of Mad1 on kinetochores (Fig. 2 and Supplementary Movie 5). In contrast, 9A-Hec1-expressing cells treated with STLC formed stable attachments as evidenced by formation of robust kinetochore–microtubule bundles and loss of kinetochore Mad1, and subsequently exited mitosis (Fig. 2 and Supplementary Movie 6). This result suggests that stabilized kinetochore–microtubule attachments, even in the absence of chromosome bi-orientation, are sufficient to satisfy the SAC and promote mitotic exit. We repeated these experiments in WT- and 9A-Hec1-expressing cells depleted of endogenous Hec1, which produced the same result (Supplementary Fig. 3). Furthermore, addition of ZM447439 to inhibit Aurora B kinase, which serves to destabilize kinetochore–microtubule attachments^35^,^36^ (and also may contribute to SAC signalling by synergizing with other SAC proteins^37^), resulted in gradual SAC satisfaction and mitotic exit (Supplementary Fig. 4). Consistent with our finding that 9A-Hec1-expressing cells satisfy rather than abrogate the SAC, the kinetics of mitotic exit in 9A-Hec1-expressing cells were similar to ZM447439-treated host (parental) HeLa cells, but not to host cells treated with 10 μM reversine, which results in rapid SAC abrogation and subsequent mitotic exit^31^ (Supplementary Fig. 4 and Supplementary Movies 7–9). Finally, to test if the mitotic exit observed in 9A-Hec1-expressing cells was indeed due to SAC satisfaction and not mitotic slippage, we expressed an mCherry-tagged version of Cyclin B in cells stably expressing 9A- and WT-Hec1 and measured loss of Cyclin B fluorescence over time (Supplementary Fig. 5). In all cells, loss of Cyclin B preceded mitotic exit, suggesting that mitotic slippage was not responsible for the observed exit from mitosis in cells expressing 9A-Hec1-GFP.

### Stable MTs induce small changes in kinetochore architecture

**Figure 3:**
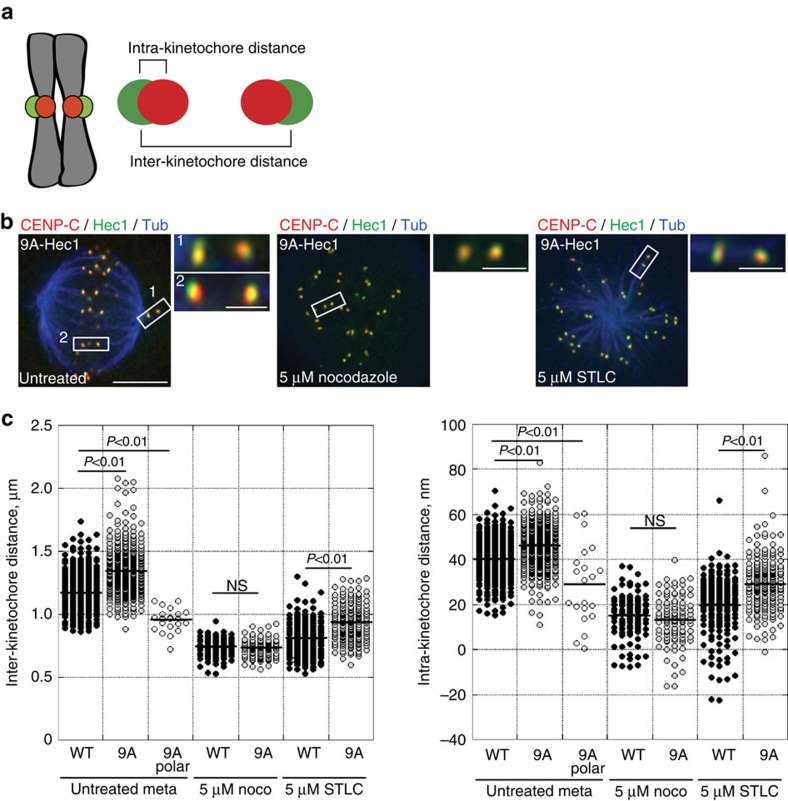
Stable attachments do not induce large-scale changes in intra-kinetochore distance in STLC-treated cells. (**a**) Cartoon depiction of inter- and intra-kinetochore distances. (**b**) Immunofluorescence images of 9A-Hec1-GFP-expressing cells. Scale bar, 5 μm. Left: untreated cell in metaphase. Boxed insets show examples of a (1) pole-proximal and (2) bi-oriented kinetochore pair. Middle: cell treated with 5 μM nocodazole. Right: cell treated with 5 μM STLC. In the insets, Hec1-GFP is shown in green, and CENP-C staining is shown in red. Scale bars are 1 μm. (**c**) Inter-kinetochore and intra-kinetochore distance measurements. Each circle represents a measured inter- or intra-kinetochore distance for a pair of sister chromatids. *n* values are listed in Table 1. NS=not significantly different, *P*≥0.01, as evaluated by Welch's two-sample *t*-tests (inter-kinetochore distances, *P*=0.52; intra-kinetochore distances, *P*=0.90).

A current model for SAC satisfaction posits that ‘stretching' of individual kinetochores, in which the outer kinetochore is pulled away from the inner kinetochore, is the critical event detected by the SAC-silencing machinery^23^,^24^. Using super-resolution co-localization microscopy (Supplementary Fig. 6), we therefore tested if kinetochores on pole-proximal chromosomes in 9A-Hec1-expressing cells (Fig. 1a) experienced such stretching before mitotic exit. We first measured intra-kinetochore distances on kinetochores of bi-oriented chromosomes to establish the ‘full' stretch level. In 9A-Hec1-expressing cells, the average distance from CENP-C (an inner kinetochore marker) to the C-terminal GFP tag on Hec1 on bi-oriented kinetochores was ∼46 nm, which was slightly larger than the distance measured in WT-Hec1-expressing cells (∼40 nm; Fig. 3 and Table 1)^38^. We then measured intra-kinetochore distances in cells treated with 5 μM nocodazole to establish the ‘rest length'. Under these conditions, the average CENP-C to Hec1-C-term distance was similar (∼14 nm) in both WT- and 9A-Hec1-expressing cells (Fig. 3c and Table 1). In the case of kinetochores on pole-proximal chromosomes in 9A-Hec1-expressing cells, the intra-kinetochore distances were slightly larger than the measured rest length (∼28 versus ∼14 nm; Fig. 3c and Table 1). These results suggest that stable kinetochore–microtubule attachments in the absence of chromosome bi-orientation generate a rearrangement of kinetochore proteins that produces a small, but significant, displacement of the outer kinetochore from the inner kinetochore. Consistent with this finding, the average intra-kinetochore distance in 9A-Hec1-expressing cells treated with STLC was ∼29 nm, compared with ∼20 nm in cells expressing WT-Hec1 (Fig. 3c and Table 1). These results demonstrate that SAC satisfaction occurs in cells that form stable kinetochore–microtubule attachments in the absence of large-scale intra-kinetochore ‘stretch.' Nevertheless, microtubule binding to kinetochores, even in cells that lack bi-oriented chromosomes, results in small, but measurable changes in kinetochore architecture.

### MT attachment silences the SAC in the absence of tension

**Figure 4:**
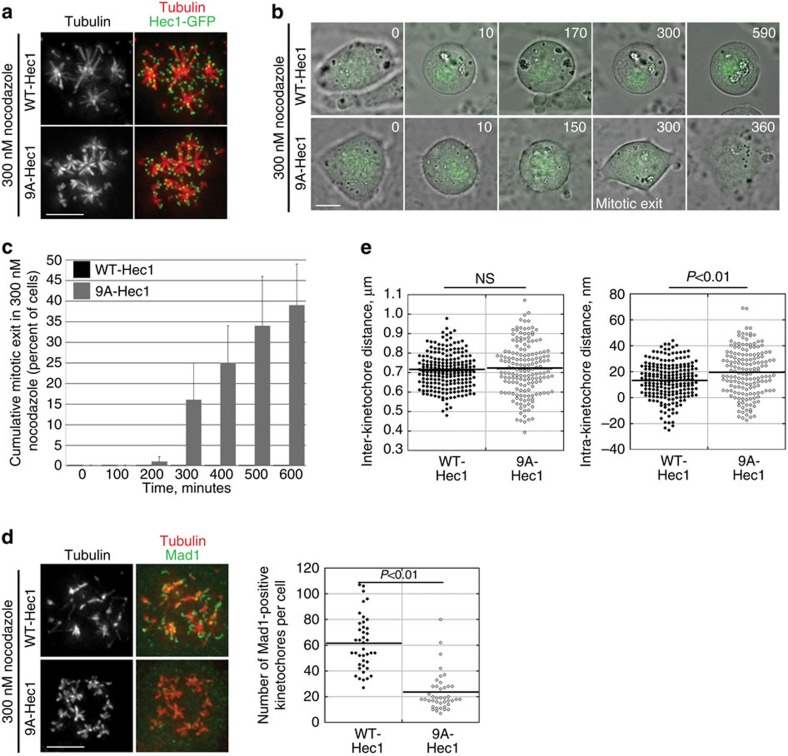
Stable kinetochore–microtubule attachment silences the SAC in the absence of spindle pole-mediated pushing or pulling forces. (**a**) Immunofluorescence images showing formation of kinetochore-associated microtubule ‘tufts' in WT- and 9A-Hec1-GFP-expressing cells. Scale bar, 5 μm. (**b**) Stills from time-lapse imaging of WT- and 9A-Hec1-GFP-expressing cells treated with 300 nM nocodazole. Shown are overlays of phase contrast and GFP images. Time is indicated in minutes, and the time of mitotic exit initiation is also indicated. Scale bar, 5 μm. (**c**) Quantification of mitotic exit in WT- and 9A-Hec1-GFP-expressing cells. Graph indicates cumulative mitotic exit at the indicated time point. Data from three independent experiments are included, *n*=156 for WT- and *n*=141 for 9A-Hec1-expressing cells. Error bars indicate s.d. (**d**) Immunofluorescence images of WT- and 9A-Hec1-GFP-expressing cells stained for Mad1. Quantification of Mad1-positive kinetochores is shown on the right. *P* value determined by Student's *t*-test. For each cell line, 41 cells were scored from three experiments. (**e**) Inter- and intra-kinetochore distance measurements for WT- and 9A-Hec1-GFP-expressing cells treated with 300 nM nocodazole. Each circle represents a measured inter- or intra-kinetochore distance for a pair of sister chromatids. *n* values are listed in Table 1. *P* values were determined from Welch's two-sample *t*-tests. NS=not significantly different, *P*=0.40.

On the basis of these results, we formulated two hypotheses. In the first hypothesis, SAC silencing in STLC-treated 9A-Hec1-expressing cells results from kinetochore tension produced via pulling forces from the attached microtubules. Support for this comes from a recent study, which found that syntelically attached kinetochores were competent to silence the SAC in *Drosophila* S2 cells, but only after polar ejection forces were experimentally increased^16^. In this case, tension arises from the opposition of kinetochore–microtubule poleward forces and chromosome-arm-mediated anti-poleward forces. In the second hypothesis, the accumulation of stable microtubules bound to the core kinetochore–microtubule attachment molecules signals for SAC satisfaction independently of the tension that results from external pulling or pushing forces. To differentiate between these two possibilities, we set out to generate conditions in which kinetochores are stably bound to microtubules in the absence of spindle pole-dependent pushing or pulling forces. We achieved this by treating cells expressing either 9A- or WT-Hec1 with a moderately low dose (300 nM) of nocodazole, which resulted in the loss of all non-kinetochore spindle microtubules but retention of kinetochore–microtubule ‘tufts,' which were comprised of short microtubule bundles attached to kinetochores (Fig. 4a). As expected, cells expressing WT-Hec1 arrested in mitosis in response to 300 nM nocodazole treatment. Strikingly, similarly treated cells expressing 9A-Hec1 exited mitosis after a delay (Fig. 4b,c and Supplementary Movies 10 and 11). Mitotic exit resulted as a consequence of SAC satisfaction as evidenced by a significant decrease in Mad1-positive kinetochores (Fig. 4d). We then tested if occupancy of kinetochore–microtubule-binding sites resulted in architectural changes in kinetochores in cells treated with 300 nM nocodazole. Under these conditions, the average distance between CENP-C and Hec1-C-term in 9A-Hec1-expressing cells was ∼20 nm, whereas the distance in WT-Hec1-expressing cells was ∼13 nm, which is equivalent to the rest length (Fig. 4e, Table 1). Together, these results suggest that stable kinetochore–microtubule-binding signals for SAC satisfaction independent of tension, and furthermore, that microtubule occupancy at kinetochores results in small, but detectable changes in kinetochore protein architecture.

## Discussion

It is well-established that formation of stable, end-on kinetochore–microtubule attachments quenches the ‘wait-anaphase' signal generated by the SAC. Although it is not yet clear how microtubule attachment turns the SAC off, recent studies have suggested that pulling forces provided by end-on attached microtubules ‘stretch' individual kinetochores, which leads to SAC satisfaction^23^,^24^. How might intra-kinetochore stretching promote SAC silencing? The prevailing model is that increasing the distance between outer kinetochore components and centromere-localized Aurora B prevents their phosphorylation^9^,^39^,^40^. This in turn, is predicted to promote to SAC silencing by tipping the balance towards kinetochore phosphatases such as PP1, whose increased kinetochore localization and activity promotes the delocalization of key checkpoint proteins, leading to SAC satisfaction^6^,^12^,^41^,^42^.

In this study, we find that stable kinetochore–microtubule attachment is sufficient to silence the SAC in the absence of large-scale changes in either inter-kinetochore or intra-kinetochore distance. Our data argue that tension, *per se*, is not a parameter read by the checkpoint machinery. How does the SAC detect and respond to stable kinetochore–microtubule attachment in the absence of ‘stretching' or tension? Two recent studies have demonstrated that end-on microtubule binding to the NDC80 complex promotes displacement of the SAC protein kinase Mps1 from kinetochores^43^,^44^. Mps1 phosphorylates the kinetochore scaffold protein KNL1 to recruit, either directly or indirectly, a suite of checkpoint proteins including Bub1, BubR1, Bub3, Mad1 and Mad2 (refs 42, 45), thus eviction of Mps1 leads to delocalization of these SAC components and subsequent SAC satisfaction. In addition, stable microtubule attachment has been shown to promote dissociation of SAC proteins through the minus-end directed dynein motor, which ‘strips' SAC components off kinetochores along spindle microtubules, thereby contributing to checkpoint silencing^8^. Finally, it is possible that stable microtubule occupancy results in biochemical and/or conformational changes in kinetochore proteins that promote the dissociation of SAC-promoting proteins such as Aurora B kinase or the SAC proteins themselves, or alternatively, in the recruitment of SAC-silencing proteins such as the phosphatase PP1 (refs 6, 12, 42, 45, 46).

Here we established experimental conditions that prevented chromosome bi-orientation and the generation of opposing pulling forces on sister kinetochores. In one scenario, cells were treated with STLC to generate monopolar spindles in which chromosomes were either syntelically or monotelically oriented. In another, cells were treated with 300 nM nocodazole to create conditions in which most spindle microtubules were depolymerized and very short kinetochore fibres were retained. In both cases, 9A-Hec1-expressing cells were able to satisfy the SAC and exit mitosis. On average, the time to mitotic exit after nuclear envelope breakdown in the presence of STLC was significantly longer than in untreated cells. We predict that the increased time required to silence the SAC results from a gradual accumulation of stable kinetochore–microtubule attachments. The relatively large distribution of times required for SAC satisfaction likely reflects a graded response of the SAC^47^, in which the rate of formation of stable kinetochore–microtubule attachments correlates to the time required for SAC silencing. It is also important to note that intra-kinetochore distances were measured in a population of fixed cells that spent a variable amount of time arrested in mitosis (that is, some cells had just entered mitosis, while others were arrested for up to several hours), which in part explains the large distribution of distances. Nevertheless, these measurements revealed a very small (∼10 nm), but statistically significant, difference in the average distance between CENP-C (an inner kinetochore protein) and the C-terminus of NDC80 (an outer kinetochore protein) in 9A- versus WT-Hec1-expressing cells. It is formally possible that this small increase in intra-kinetochore distance triggers SAC silencing. However, it is difficult to envision a scenario in which moving the outer kinetochore away from the inner kinetochore by such a small distance is sufficient to limit the access of Aurora B kinase, which is proposed to emanate as a gradient from the centromere, to outer kinetochore substrates. We propose instead that the small change in intra-kinetochore distance results from alterations in overall kinetochore architecture that are a consequence of stable microtubule binding. In support of this notion, a recent study from the Salmon lab demonstrated in HeLa cells that intra-kinetochore distances increased by ∼10 nm on average from late prometaphase to metaphase, which represents the transition from SAC activation to SAC silencing^25^. Interestingly, in this study, the authors demonstrated that the major drop in Aurora B kinase activity, measured using phospho-specific Hec1 antibodies, occurred at this transition from late prometaphase to metaphase^25^. Thus, the large decrease in Aurora B kinase activity at kinetochores does not coincide with a large-scale change in intra-kinetochore distance. This suggests, together with the findings from our study, that the SAC is not silenced by intra-kinetochore stretch and the spatial re-positioning of outer kinetochore components in relation to the inner kinetochore. Instead, it is likely that SAC silencing occurs owing to a cascade of biochemical and conformational changes within kinetochore proteins and protein complexes that are triggered by stable, end-on microtubule binding that lead to SAC protein eviction. How stable attachment signals such architectural changes that ultimately silence the SAC is not known, but likely involves conformational changes within both inner and outer kinetochore proteins, including CENP-C, CENP-T, KNL1 and the NDC80 complex^25^,^38^,^48^,^49^,^50^.

## Methods

### Cell culture, transfections and generation of cell lines

Stable cell lines expressing inducible WT-Hec1-GFP or 9A-Hec1-GFP were generated from a FlpIn T-REx HeLa host cell line (a gift from Stephen Taylor, University of Manchester, Manchester, England). Cells were grown to 50% confluence in DMEM supplemented with 10% fetal bovine serum (FBS), 1% penicillin/streptomycin, and 2 mM L-glutamine at 37 °C in 5% CO_2_. Cells were transfected with 2.4 μg pOG44 recombinase-containing plasmid and 0.3 μg pcDNA5.FRT.TO-WT- or 9A-Hec1-GFP containing plasmids with Fugene HD (Promega). The pcDNA5.FRT.TO-Hec1 plasmids were generated through PCR amplification of WT- and 9A-Hec1-GFP fragments and cloned into a pcDNA5.FRT.TO vector through In-Fusion cloning. After 48 h, cells were switched to media containing 100 μg ml^−1^ hygromycin (EMD Millipore) and grown in this selection media for 2 weeks. Hygromycin-resistant foci were expanded and examined for inducible Hec1-GFP expression^51^. Gene expression was induced with 1 μg ml^−1^ doxycycline (Sigma-Aldrich) for 30 h. For silence and rescue experiments in which endogenous Hec1 was depleted, 7 μl of a 20 μM stock solution of Cy-5-labelled, human-specific Hec1 siRNA targeted to the 5′ UTR (5′-CCCUGGGUCGUGUCAGGAA-3′) was added to 150 μl of OptiMem (Invitrogen). Concurrently, 6 μl of Oligofectamine (Invitrogen) was added to 150 μl of OptiMem. Samples were incubated in 1.7 ml microfuge tubes at room temperature for 5 min. Contents of the two tubes were combined and incubated for an additional 20 min before adding to each well of a 6-well dish containing 50% confluent HeLa cells in 1 ml OptiMem plus 10% FBS. The following day an additional 1 ml OptiMem/FBS plus 2 μg ml^−1^ doxycycline was added to each well. Coverslips were processed at 48 h. mCherry-Cyclin B was transiently expressed using Fugene 6 Transfection Reagent (Promega) lipid transfection agent. Fugene (5 μl) was added to 95 μl of OptiMEM for each well of a 6-well dish. Following a 5 min incubation at room temperature, 750 ng mCherry-Cyclin B (a gift from Jonathon Pines, The Gurdon Institute, Cambridge, UK) was added. After 20 min at room temperature, the solution was added dropwise to 2 ml Optimem plus 10% FBS. Cells were imaged at 24 h post transfection.

### Western blotting and quantification

To determine Hec1 protein expression levels, cells were grown in 25 cm^2^ flasks to 80% confluency. Cells were collected from the flasks with trypsin, pelleted in a tabletop centrifuge and raised in cold 1X PBS (140 mM NaCl, 2.5 mM KCl, 1.6 mM KH_2_PO_4_, 15 mM Na_2_HPO_4_, pH 7.0), 2 mM dithiothreitol and protease inhibitor cocktail (Thermo). Cells were sonicated on ice (Ultra Sonic Device) and lysates were clarified by centrifugation. Lysate protein concentrations were quantified by Bradford Assay and then boiled for 1 min with 1X SDS Sample Buffer. Protein samples (30 μg) were run on 12% SDS-polyacrylamide gels and transferred to polyvinylidene difluoride membrane (Millipore). Blots were probed for Hec1 with mouse anti-Hec1 antibodies (Novus Biologicals, GTX70268) at a dilution of 1:2,000. Anti-α-tubulin antibodies (Sigma, T6199) were used at a dilution of 1:6,000 for a loading control. Primary antibodies were detected using horseradish peroxidase-conjugated-anti-mouse secondary antibody at a dilution of 1:10,000 (Gene Script Corp., A00160) and visualized via chemiluminescence (Thermo Scientific). Chemiluminescent images were obtained on an ImageQuant LAS 500 imager. Bands were background subtracted and quantified using Metamorph software.

### Immunofluorescence

Before fixation and lysis, cells were rinsed in PHEM Buffer (60 mM PIPES, 25 mM HEPES, 10 mM EGTA, 8 mM MgSO_4_, pH 7.0) pre-warmed to 37 °C. For fixed-cell analysis of inter- and intra-kinetochore distance measurements, cells were fixed in 4% paraformaldehyde at 37 °C for 20 min and subsequently lysed in PHEM buffer+0.5% Triton X-100 at 37 °C for 5 min. For all other immunofluorescence experiments, cells were lysed in PHEM buffer+0.5% Triton X-100 at 37 °C for 5 min, followed by fixation in 4% paraformaldehyde at 37 °C for 20 min. Cells were then rinsed 3 × 15 min in PHEM+0.05% Triton X-100 and blocked for 1 h at room temperature with 10% boiled donkey serum (BDS) in PHEM. Primary antibodies were diluted in 5% BDS in PHEM as follows: mouse anti-Hec1-9G3, 1:2,500 (Novus Biologicals, GTX70268), rabbit anti-Mad1, 1:200 (GeneTex, GTX109519), rabbit anti-CENP-A, 1:400 (Cell Signaling, 2186S), guinea pig anti-CENP-C, 1:1,000 (Medical and Biological Laboratories, PD030), rabbit anti-GFP, 1:500 (Invitrogen, A6455), mouse anti-α-tubulin, 1:300 (Sigma, T6199) and mouse anti-BubR1, 1:200 (Millipore, MAB3612). Cells were incubated with primary antibodies overnight at 4 °C, and then rinsed 3 × 15 min in PHEM+0.05% Triton X-100. Cells were incubated with secondary antibodies conjugated to Alexa488 or Alexa647 (Jackson ImmunoResearch Laboratories, 715-545-150 and 715-605-150 respectively) or Alexa568 (Abcam, 175470) diluted 1:300 in 5% BDS in PHEM for 45 min at room temperature. Cells were rinsed 3 × 5 min in PHEM+0.05% Triton X-100 and subsequently incubated in 4′,6-diamidino-2-phenylindole diluted to 2 ng ml^−1^ for 1 min at room temperature. Cells were rinsed with PHEM+0.05% Triton X-100 4 × 5 min, rinsed once with PHEM, and mounted onto slides using the following mounting media: 20 mM Tris, pH 8.0, 0.5% *N*-propyl gallate, and 90% glycerol. Coverslips were sealed to the slides using fingernail polish. Before fixation cells were treated as indicated with 5 μM STLC (Tocris) or with 5 μM or 300 nM nocodazole (Tocris).

### Image acquisition and analysis

Images were acquired on a DeltaVision Personal DV (Applied Precision) imaging system equipped with a CoolSNAP HQ2 (Photometrics/Roper Scientific) camera with a 60X/1.42 NA PlanApochromat objective and SoftWorx acquisition software (Applied Precision). Images for fixed-cell experiments were acquired as z-stacks at 200 nm intervals. Kinetochores were identified by Hec1-GFP position, and fluorescence intensities of proteins of interest were determined using custom MATLAB software (‘Speckle Tracker'; Mathworks, Natlick, MA) written by Drs Xiaohou Wan and Ted Salmon. Fluorescence intensities were normalized to the level of GFP-fusion protein expression using the GFP fluorescence intensity. To determine the number of Mad1-positive kinetochores, kinetochore signals were identified by GFP-Hec1 localization and scored from deconvolved images. Inter- and intra-kinetochore distance measurements were performed on sister kinetochore pairs that resided in a single focal plane. The centroids of GFP-Hec1 and antibody-labelled CENP-C were determined by custom-written MATLAB software (provided by Drs Xiaohou Wan and Ted Salmon). Inter-kinetochore distances were calculated using the centroids of the GFP signal (C-terminal GFP-Hec1, labelled with anti-GFP antibodies to increase fluorescence signal) on each of the two kinetochores in a sister kinetochore pair. Intra-kinetochore distances (Hec1-GFP to CENP-C) were calculated as one-half the difference of the distance between outer kinetochore centroids (Hec1-GFP) and inner kinetochore centroids (CENP-C)^38^. Before carrying out these measurements, we carried out control experiments, in which inter- and intra-kinetochore distances were measured from kinetochores stained with Hec1-9G3 antibodies followed by simultaneous staining with Alexa488 and Alexa568 secondary antibodies (Supplementary Fig. 6). Live cell images were acquired on the DeltaVision imaging system described above using a 60X/1.42 NA PlanApochromat or a 40X/0.75NA UPlanFL objective. Cells were imaged in a 37 °C environmental chamber in Leibovitz's L-15 media (Gibco) supplemented with 10% FBS, 7 mM HEPES and 4.5 g/l D-glucose (pH 7.0). As indicated in the text, live cell experiments were carried out using the following drug concentrations: 5 μM STLC (Tocris), 5 μM or 300 nM nocodazole (Tocris), 2 mM ZM447439 (Tocris) and 10 μM reversine (Sigma-Aldrich). For determining mitotic transit time, cells were scored only if they entered mitosis during imaging. To determine if cells underwent mitotic slippage, HeLa cells stably expressing WT- or 9A-Hec1-GFP were transfected with an mCherry-Cyclin B expression vector^52^. Cells were time-lapse imaged and total mCherry cell fluorescence was measured over time using SoftWorx analysis software.

### Statistical analysis

Most statistical comparisons were made using two-tailed Student's *t*-tests, as indicated in the figure legends. Normality was determined through Anderson–Darling tests for normality. Kinetochore distance measurements were compared using Welch's two-sample *t*-tests. In addition, linear mixed effects models, appropriate for nested data (kinetochore pairs within cells, within experiments) were used for kinetochore distance comparisons. The experimental data were analysed in the R statistical computing environment^53^,^54^. Restricted maximum likelihood methods were used, as implemented in the R package lme4 (ref. 55) to fit the models. Fitted values, standard deviations, and standard errors were calculated based on the mixed effects models including omission of random effects. The values obtained from this analysis are shown in Supplementary Table 1. R code used for analysis is available on request.

## Additional information

**How to cite this article:** Tauchman, E. C. *et al.* Stable kinetochore–microtubule attachment is sufficient to silence the spindle assembly checkpoint in human cells. *Nat. Commun.* 6:10036 doi: 10.1038/ncomms10036 (2015).

## Supplementary Material

Supplementary Information Supplementary Figures 1-6 and Supplementary Table 1

Supplementary Movie 1 HeLa cells expressing WT-Hec1-GFP enter anaphase with fully aligned chromosomes. A WT-Hec1-GFP expressing cell was imaged using a 60X oil-immersion objective at 3 min intervals. The cell shown enters anaphase only after all chromosomes are aligned. Scale bar is 10 μm.

Supplementary Movie 2 HeLa cells expressing 9A-Hec1-GFP enter anaphase with unaligned chromosomes. A 9A-Hec1-GFP expressing cell was imaged using a 60X oil-immersion objective at 3 min intervals. The cell shown enters anaphase with unaligned chromosomes. Scale bar is 10 μm.

Supplementary Movie 3 HeLa cells expressing WT-Hec1-GFP exhibit a sustained mitotic arrest in the presence of 5 μM nocodazole. Cells were imaged using a 60X oil-immersion objective at 5 min intervals. DIC image is overlaid with the WT-Hec1-GFP image. The cell shown enters mitosis and arrests for 690 min, at which time imaging was terminated. Scale bar is 10 μm.

Supplementary Movie 4 HeLa cells expressing 9A-Hec1-GFP exhibit a sustained mitotic arrest in the presence of 5 μM nocodazole. Cells were imaged using a 60X oil-immersion objective at 5 min intervals. DIC image is overlaid with the 9A-Hec1-GFP image. The cell shown enters mitosis and arrests for 750 min, at which time imaging was terminated. Scale bar is 10 μm.

Supplementary Movie 5 HeLa cells expressing WT-Hec1-GFP exhibit a sustained mitotic arrest in the presence of 5 μM STLC. Cells were imaged using a 60X oil-immersion objective at 5 min intervals. DIC image is overlaid with the WT-Hec1-GFP image. The cell shown enters mitosis and arrests for 670 min, at which time imaging was terminated. Scale bar is 10 μm.

Supplementary Movie 6 HeLa cells expressing 9A-Hec1-GFP exit mitosis in the presence of 5 μM STLC. Cells were imaged using a 60X oil-immersion objective at 5 min intervals. DIC image is overlaid with the WT-Hec1-GFP image. The cell shown enters mitosis and subsequently exits mitosis after 170 min. Scale bar is 10 μm.

Supplementary Movie 7 Host HeLa Flp-In cells exhibit a sustained mitotic arrest in the presence of 5 μM STLC. Host cells (not containing Hec1-GFP) were imaged using a 40X objective at 5 min intervals. The cell shown (DIC image) enters mitosis and arrests for 665 min, at which time imaging was terminated. Scale bar is 10 μm.

Supplementary Movie 8 Host HeLa Flp-In cells treated with 5 μM STLC and 2 μM ZM447439 satisfy the SAC and exit mitosis after a delay. Host cells (not containing Hec1-GFP) were treated 2 μM ZM447439 to inhibit Aurora B kinase. These cells were imaged using a 40X objective at 5 min intervals. The cell shown (DIC image) enters mitosis and subsequently exits after 320 min. Scale bar is 10 μm.

Supplementary Movie 9 Host HeLa Flp-In cells treated with 5 μM STLC and 10 μM Reversine do not exhibit a mitotic delay and abrogate the SAC. Host cells (not containing Hec1-GFP) were treated 10 μM Reversine to inhibit Mps1 kinase. These cells were imaged using a 40X objective at 5 min intervals. The cell shown (DIC image) enters mitosis and subsequently exits after 45 min. Scale bar is 10 μm.

Supplementary Movie 10 HeLa cells expressing WT-Hec1-GFP exhibit a sustained mitotic arrest in the presence of 300 nM nocodazole. Cells were imaged using a 60X oil-immersion objective at 5 min intervals. DIC image is overlaid with the WT-Hec1-GFP image. The cell shown enters mitosis and arrests for 600 min, at which time imaging was terminated. Scale bar is 10 μm.

Supplementary Movie 11 HeLa cells expressing 9A-Hec1-GFP exit mitosis in the presence of 300 nM nocodazole. Cells were imaged using a 60X oil-immersion objective at 5 min intervals. DIC image is overlaid with the WT-Hec1-GFP image. The cell shown enters mitosis and subsequently exits mitosis after 310 min. Scale bar is 10 μm.

## Figures and Tables

**Table 1:** Summary of mean values of inter- and intra-kinetochore distances measured in HeLa cells expressing WT- or 9A-Hec1-GFP.

		**Inter-kinetochore distance, μm**	**Intra-kinetochore distance, nm**	**N kinetochores/N cells**
WT-Hec1	Aligned	1.17 (0.08)	40.2 (4.9)	450/30
9A-Hec1	Aligned	1.34 (0.11)	46.2 (5.6)	414/30
9A-Hec1	Polar	0.95 (0.05)	28.9 (10.2)	21/14
WT-Hec1	5 μM noco	0.73 (0.04)	15.1 (5.6)	101/15
9A-Hec1	5 μM noco	0.73 (0.04)	13.2 (6.0)	104/15
WT-Hec1	5 μM STLC	0.81 (0.08)	20.0 (7.0)	194/30
9A-Hec1	5 μM STLC	0.93 (0.08)	29.4 (6.8)	258/30
WT-Hec1	300 nM noco	0.71 (0.05)	12.6 (8.7)	214/30
9A-Hec1	300 nM noco	0.73 (0.08)	20.0 (10.4)	163/30

Values indicate mean distances; numbers in parentheses indicate s.e.m. The first three rows display mean inter- and intra-kinetochore distances of aligned sister kinetochore pairs in cells expressing either WT- or 9A-Hec1-GFP with no drug treatment and pole-proximal kinetochore pairs in cells expressing 9A-Hec1-GFP with no drug treatment. All other conditions are indicated.
