# Supplementary material for: Stable kinetochore–microtubule attachment is sufficient to silence the spindle assembly checkpoint in human cells
Source: Nat Commun. 2015 Dec 1;6:10036. doi: 10.1038/ncomms10036 (PMC4686653; doi:10.1038/ncomms10036)
Supplement: Supplementary Information — Supplementary Figures 1-6 and Supplementary Table 1 [file ncomms10036-s1.pdf]

Supplementary Information

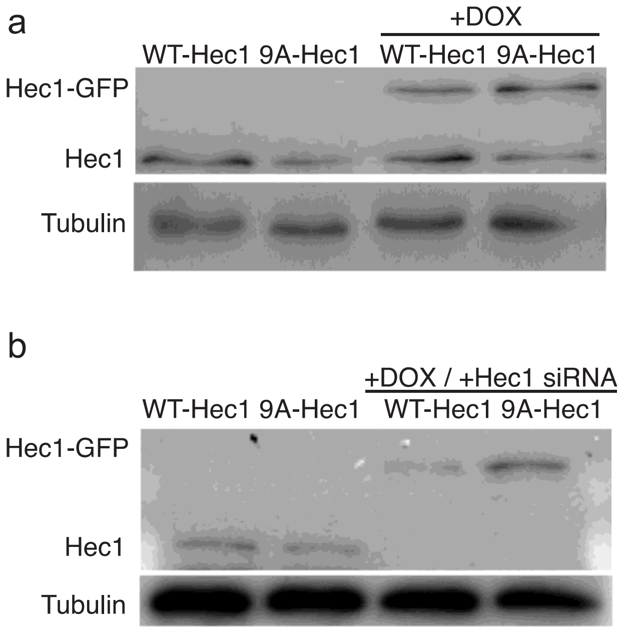

Supplementary Figure 1. Western blot analysis of HeLa cells stably expressing WT- and 9A-Hec1-GFP. (a) Western blot showing endogenous Hec1 and exogenous WT- and 9A-Hec1-GFP in HeLa Flp-In cell lines. The first two lanes contain clarified cell lysates from uninduced cells; the last two lanes contain lysates from cells induced with doxycycline to express WT- or 9A-Hec1-GFP. Band intensities were quantified and averaged over three experiments. In doxycyline-treated cells, exogenous WT-Hec1-GFP expression levels were ~92% of endogenous Hec1 levels, and exogenous 9A-Hec1-GFP expression levels were ~106% of endogenous Hec1. Comparison of the expression levels of doxycyline-treated cells revealed that WT-Hec1-GFP levels were ~82% of 9A-Hec1-GFP levels over the entire cell population. By analyzing individual cells, we determined that, on average, a higher percentage of the doxycycline-induced 9A-Hec1-GFP cells were expressing the construct compared to the WT population. However, kinetochore fluorescence intensity measurements revealed nearly identical levels of kinetochore-associated WT- and 9A-Hec1-GFP. (b) Western blot showing endogenous Hec1 and exogenous WT- and 9A-Hec1-GFP in HeLa Flp-In cell lines treated with Hec1 siRNA. The first two lanes contain clarified cell lysates from uninduced cells; the last two lanes contain lysates from cells treated with doxycycline to induce expression of WT- or 9A-Hec1-GFP and depleted of endogenous Hec1. For these “knock-out / knock-in” experiments, similar to those above, we found that a higher percentage of the doxycycline-induced 9A-Hec1-GFP cells were expressing the stable construct compared to the WT population, however, kinetochore fluorescence intensity measurements revealed nearly identical levels of kinetochore-associated WT- and 9A-Hec1-GFP in individual cells.

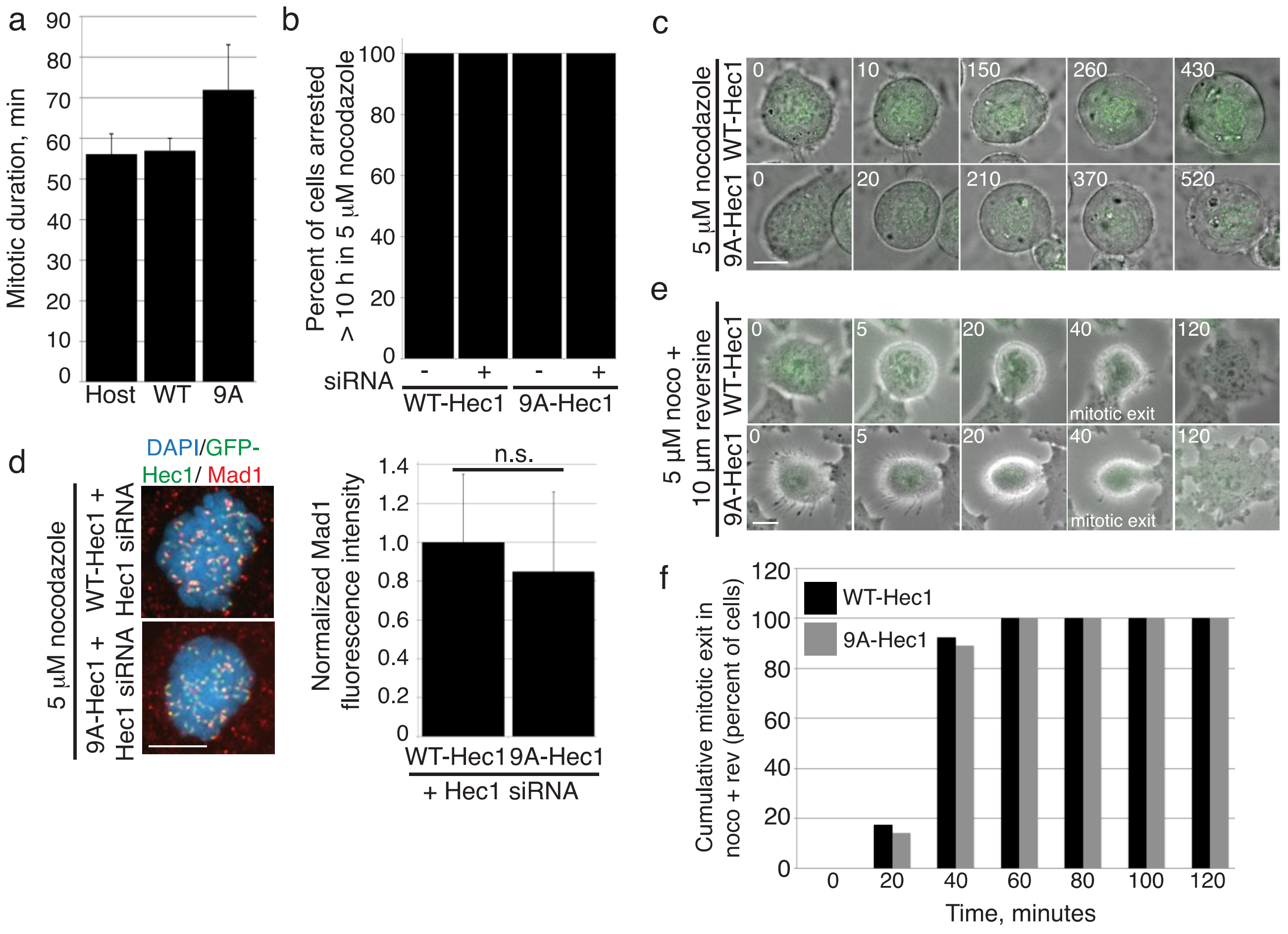

Supplementary Figure 2. SAC signaling is functional in cells expressing WT- and 9A-Hec1-GFP. (a) Graph indicating mitotic transit times for the host HeLa cell line, WT-Hec1-GFP expressing cells, and 9A-Hec1-GFP expressing cells. Mitotic transit time was scored from cell rounding to anaphase onset. Bars indicate standard deviation. n=100 cells per condition. (b) Graph indicating the percent of cells arrested for greater than 10 hours in 5 μM nocodazole. For all cell lines shown, no cells were observed to exit mitosis. n=100 cells for WT/no siRNA; n=49 cells for WT/siRNA; n=100 cells for 9A/no siRNA; n=47 cells for 9A/siRNA. (c) Stills from time-lapse imaging of WT- and 9A-Hec1-GFP expressing cells treated with 5 μM nocodazole. Shown are overlays of phase-contrast and GFP images. Time is indicated in minutes. Scale bar is 10 μm. (d) Immunofluorescence images and quantification of kinetochore fluorescence intensities of Mad1 in WT- and 9A-Hec1-GFP-expressing cells depleted of endogenous Hec1. Error bars indicate standard deviation. For each cell line, 3 experiments were performed. n=299 kinetochores for WT- and n=291 kinetochores for 9A-Hec1-GFP expressing cells. Scale bar is 5 μm. n.s. = not significantly different, p=0.15, as evaluated by Student's t-test. (e) Representative images from time-lapse movies of WT- and 9A-Hec1-GFP expressing cells treated with nocodazole and reversine. Shown are overlays of phase-contrast and GFP images. Time is indicated in minutes. Scale bar is 10 μm. (f) Quantification of mitotic exit for the indicated cell lines treated with 5 μm nocodazole and 10 μm reversine. Bars indicate cumulative mitotic exit at the indicated time point. Shown is one representative experiment, n=50 cells for WT- and n=21 cells for 9A-Hec1-GFP expressing cells.

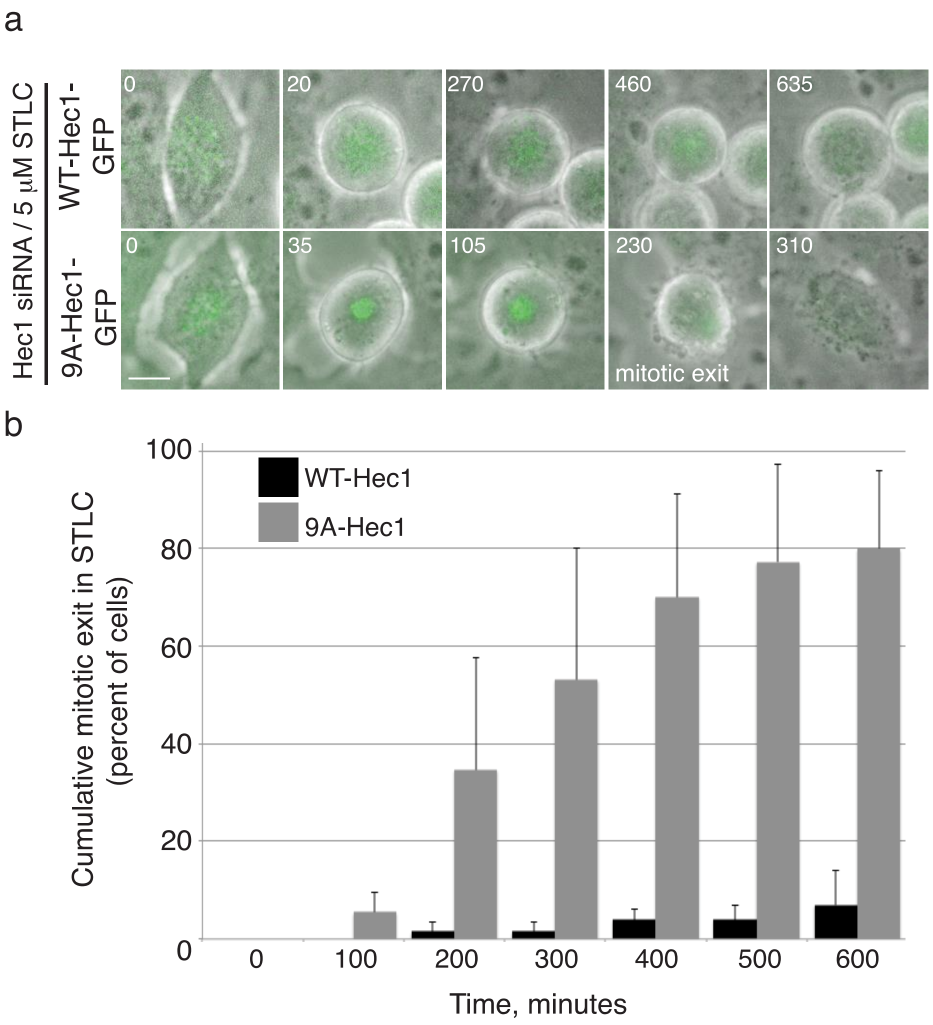

Supplementary Figure 3. Stable kinetochore-microtubule attachment is sufficient to satisfy the SAC in cells expressing 9A-Hec1-GFP and depleted of endogenous Hec1. (a) Stills from time-lapse imaging of STLC-treated WT- and 9A-Hec1-GFP expressing cells depleted of endogenous Hec1. Shown are overlays of phase-contrast and GFP images. Time is indicated in minutes. Scale bar is 10 μm. (b) Quantification of mitotic exit for WT- and 9A-Hec1-GFP expressing cells depleted of endogenous Hec1. Graph indicates cumulative mitotic exit at the indicated time point. Data from two independent experiments are included, n=111 for WT- and n=85 for 9A-Hec1-GFP expressing cells. Error bars indicate standard deviation.

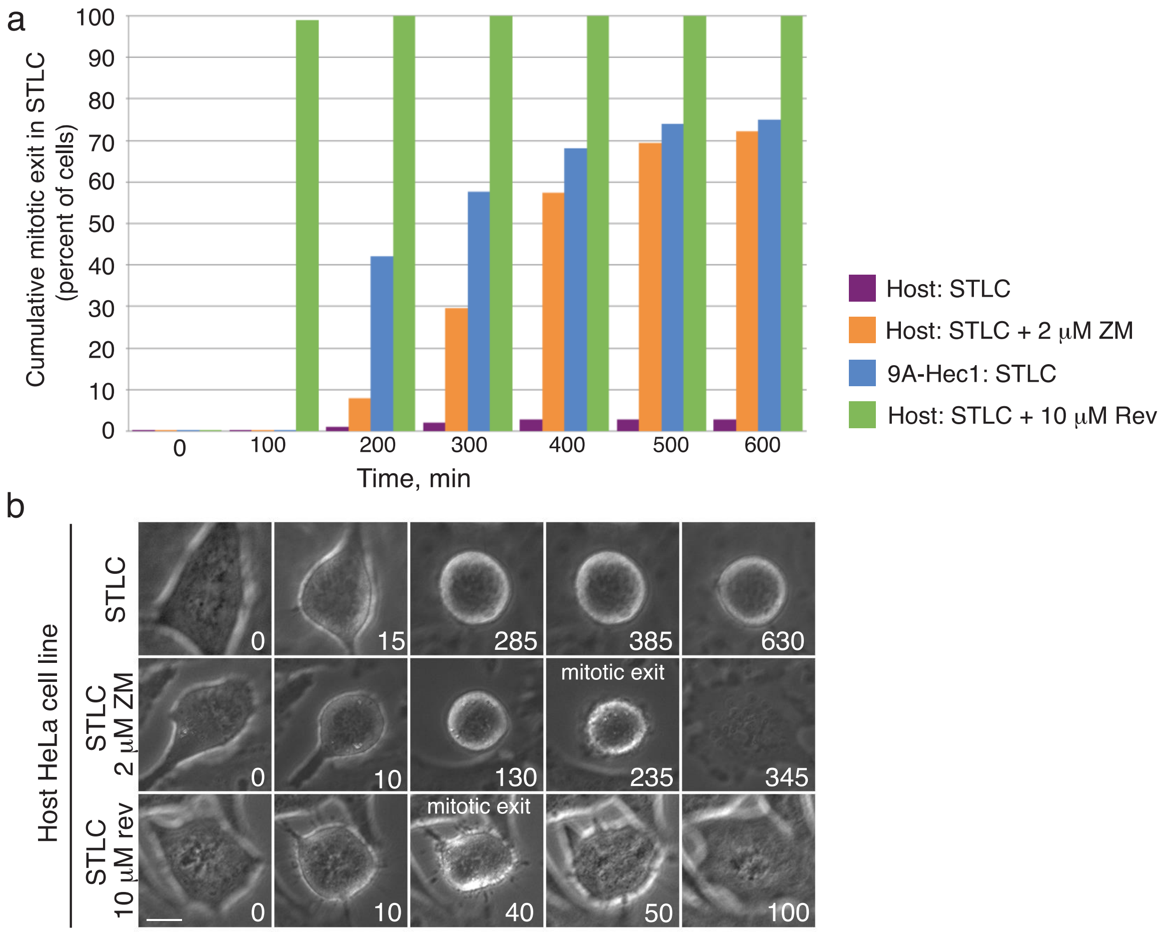

Supplementary Figure 4. Formation of hyper-stable kinetochore-microtubule attachments results in SAC silencing, not SAC abrogation. (a) Quantification of mitotic exit for the indicated cell lines. Bars indicate cumulative mitotic exit at the indicated time point. Shown is one representative experiment in which 100 cells were measured per condition. (b) Representative images from time-lapse movies. Time is indicated in minutes. Scale bar is 10 μm.

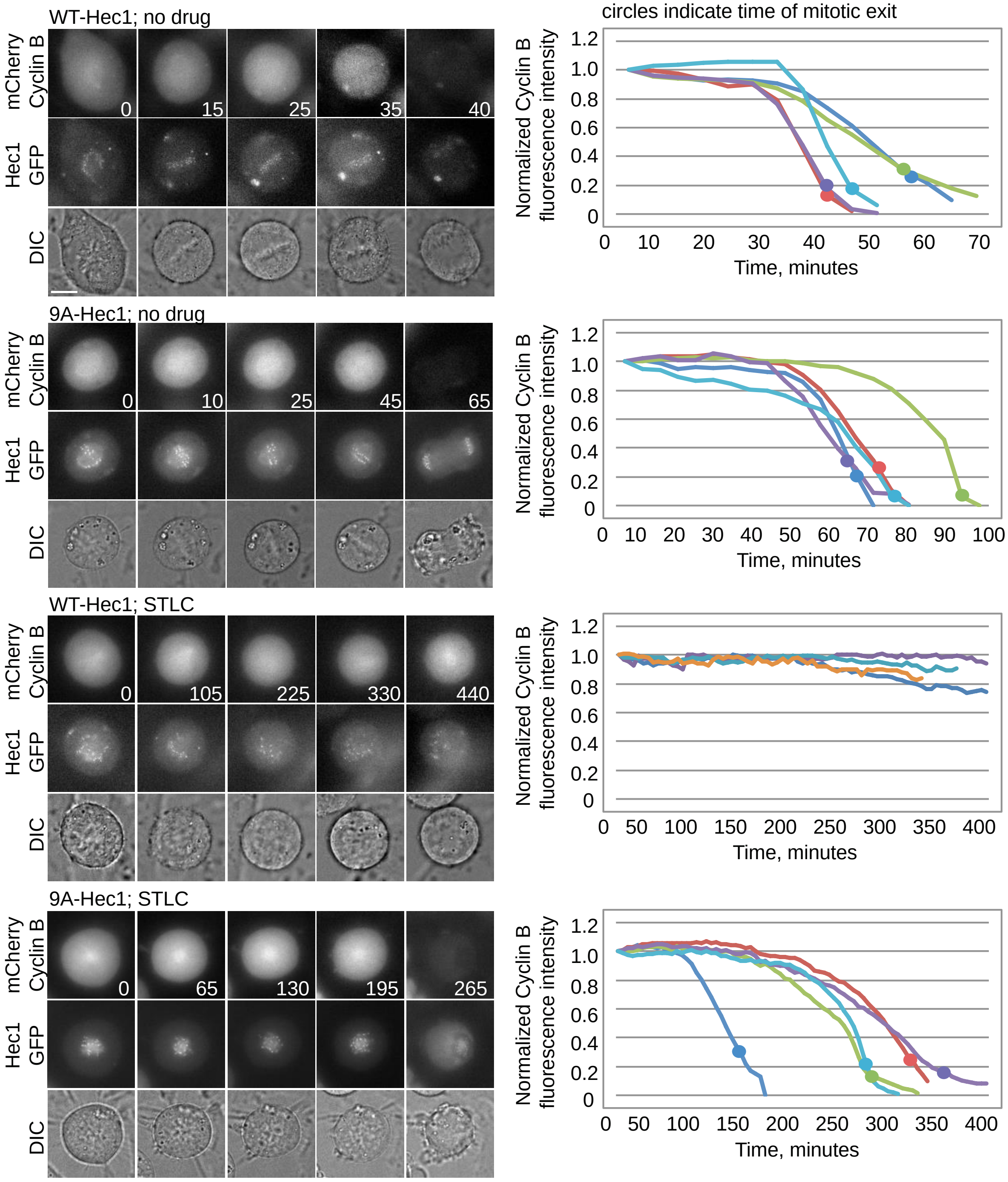

Supplementary Figure 5. Mitotic exit in STLC-treated, 9A-Hec1-GFP expressing cells is not due to mitotic slippage. The indicated cells were transfected with mCherry-Cyclin B and imaged over time. Stills from time-lapses are shown on the left. Time is indicated in minutes. Scale bar is 10 μm. Shown on the right are graphs indicating normalized mCherry-Cyclin B whole-cell fluorescence intensity over time. Lines indicate representative individual cells. At least 12 cells were analyzed for each condition. Mitotic exit times are indicated by filled circles. In all cells, mitotic exit only occurred after loss of mCherry-Cyclin B fluorescence.

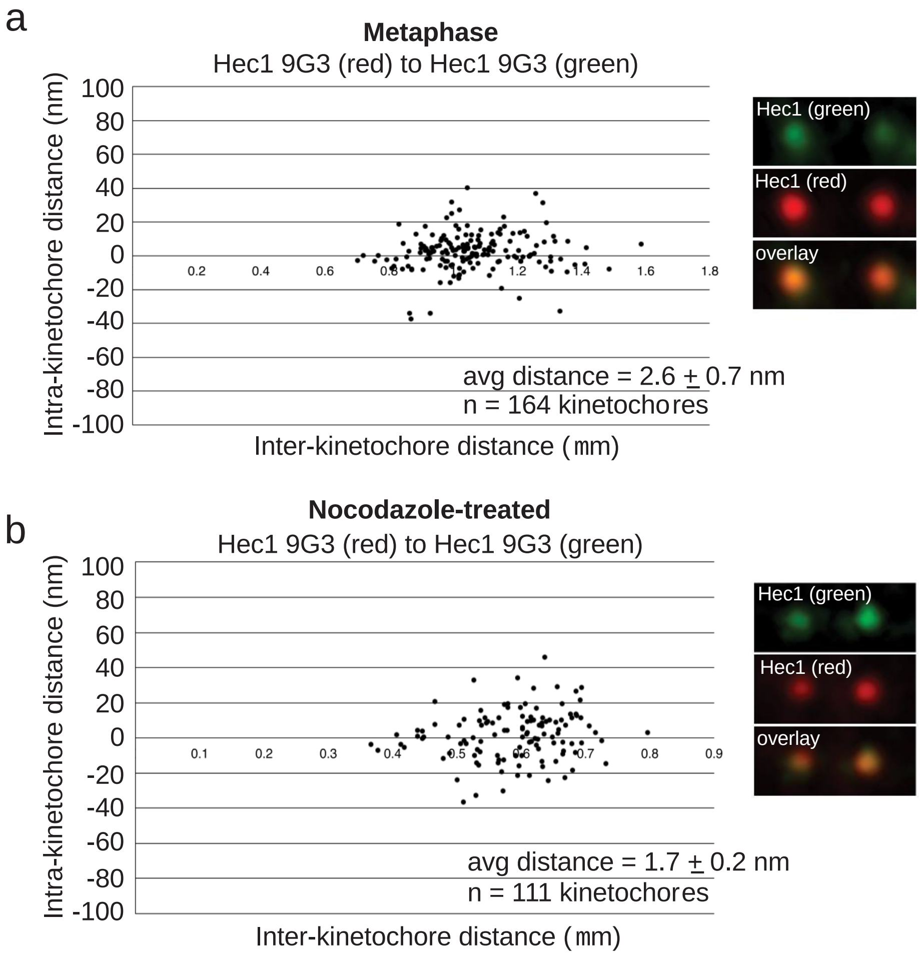

Supplementary Figure 6. Intra-kinetochore distance measurements. Cells were fixed and incubated with primary antibodies directed against Hec1 (Hec1 9G3) followed by incubation with two secondary antibodies: donkey anti-mouse-Alexa488 and donkey anti-mouse-Alexa568. Intra-kinetochore distances were measured in both untreated metaphase cells (a) and nocodazole-treated cells (b) n values are shown on the graphs; data are from 3 independent experiments.

		Inter-kinetochore distance (μm)		Intra-kinetochore distance (nm)		N kinetochores/ N cells
WT-Hec1	aligned	1.16	(0.01)	39.9	(0.4)	450/30
9A-Hec1	aligned	1.34	(0.01)	45.9	(0.4)	414/30
9A-Hec1	polar	0.92	(0.01)	28.1	(2.0)	21/14
WT-Hec1	5 μM noco	0.73	(0.01)	14.7	(0.7)	101/15
9A-Hec1	5 μM noco	0.73	(0.01)	12.9	(0.7)	104/15
WT-Hec1	5 μM STLC	0.80	(0.01)	19.8	(0.5)	194/30
9A-Hec1	5 μM STLC	0.93	(0.01)	29.2	(0.5)	258/30
WT-Hec1	300 nM noco	0.71	(0.01)	12.8	(0.5)	214/30
9A-Hec1	300 nM noco	0.72	(0.01)	19.7	(0.6)	163/30

Supplementary Table 1. Inter- and intra-kinetochore distances measured in HeLa cells expressing WT- or 9A-Hec1-GFP displayed as fitted values determined by a linear mixed effects model. Values indicate fitted distances calculated using a linear mixed effects model, which is appropriate for nested data, such as kinetochore pairs within cells, within experiments (see Methods). Numbers in parentheses indicate standard error of the mean. The first three rows display calculated inter- and intra-kinetochore distances of aligned sister kinetochore pairs in cells expressing either WT- or 9A-Hec1-GFP with no drug treatment and pole-proximal kinetochore pairs in cells expressing 9A-Hec1-GFP with no drug treatment. All other conditions are indicated.
